# ARID1A loss activates MAPK signaling via DUSP4 downregulation

**DOI:** 10.1186/s12929-023-00985-5

**Published:** 2023-12-09

**Authors:** Jayaprakash Mandal, Zheng-Cheng Yu, Ie-Ming Shih, Tian-Li Wang

**Affiliations:** 1 Department of Pathology, Johns Hopkins University School of Medicine, Baltimore, MD USA; 2 Department of Gynecology and Obstetrics, Johns Hopkins University School of Medicine, Baltimore, MD USA; 3 Department of Oncology, Sidney Kimmel Comprehensive Cancer Center, Johns Hopkins University School of Medicine, Baltimore, MD USA

**Keywords:** ARID1A, DUSP4, MAPK, Chromatin remodeling, Therapeutics

## Abstract

**Background:**

*ARID1A*, a tumor suppressor gene encoding BAF250, a protein participating in chromatin remodeling, is frequently mutated in endometrium-related malignancies, including ovarian or uterine clear cell carcinoma (CCC) and endometrioid carcinoma (EMCA). However, how *ARID1A* mutations alter downstream signaling to promote tumor development is yet to be established.

**Methods:**

We used RNA-sequencing (RNA-seq) to explore transcriptomic changes in isogenic human endometrial epithelial cells after deleting *ARID1A*. Chromatin immunoprecipitation sequencing (ChIP-seq) was employed to assess the active or repressive histone marks on DUSP4 promoter and regulatory regions. We validated our findings using genetically engineered murine endometroid carcinoma models, human endometroid carcinoma tissues, and in silico approaches.

**Results:**

RNA-seq revealed the downregulation of the MAPK phosphatase dual-specificity phosphatase 4 (DUSP4) in ARID1A-deficient cells. ChIP-seq demonstrated decreased histone acetylation marks (H3K27Ac, H3K9Ac) on DUSP4 regulatory regions as one of the causes for DUSP4 downregulation in ARID1A-deficient cells. Ectopic DUSP4 expression decreased cell proliferation, and pharmacologically inhibiting the MAPK pathway significantly mitigated tumor formation in vivo.

**Conclusions:**

Our findings suggest that ARID1A protein transcriptionally modulates DUSP4 expression by remodeling chromatin, subsequently inactivating the MAPK pathway, leading to tumor suppression. The ARID1A-DUSP4-MAPK axis may be further considered for developing targeted therapies against *ARID1A*-mutated cancers.

**Supplementary Information:**

The online version contains supplementary material available at 10.1186/s12929-023-00985-5.

## Background

Endometrial cancer, a common female malignancy, originates in the inner layer of the uterus, referred to as the endometrium. Endometrial cancer is the six most common cancer in women worldwide, and the global prevalence of endometrial cancer has significantly increased with 417,000 new cases recorded in 2020 [1]. It is estimated that approximately 3% of women will be diagnosed with this disease in their lifetime,. A staggering 132% surge in its incidence has occurred in the past three decades, an outcome associated with the increased prevalence of risk factors, notably aging and obesity [2]. A significant disparity is evident in endometrial cancer mortality rates between women from low- and middle-income countries and those from high-income countries. This discrepancy may be attributed to limited access to timely and evidence-based health care. In the United States, African American women as compared to while women are reported to have a higher likelihood of developing an aggressive subtype of endometrial cancer [3, 4].

AT-rich interaction domain 1A (*ARID1A*) is a tumor suppressor gene which encodes a protein, BAF250, that is associated with chromatin remodeling. It is the most frequently mutated gene among chromatin remodeling genes. It forms a part of the SWItch/Sucrose Non-fermentable (SWI/SNF) complex, specifically the canonical BAF (cBAF) variant. The SWI/SNF complex enables DNA accessibility to nuclear proteins and their complexes. This complex binds to various regions in the genome, including distal enhancer regions, promoter regions, and CCCTC-binding factor (CTCF)-binding sites (for review [5]). Approximately 6% of human cancers have *ARID1A* mutations that cause its inactivation, and these mutations are seen most frequently in clear cell ovarian cancers (~ 50%), endometrial cancers (~ 37%), gastric cancers (20–30%), and bladder cancers (~ 20%) s) [6–10]. *ARID1A* mutations in endometrial endometrioid carcinoma patients are associated with poor prognosis [11]. A synthetic lethal treatment approach may be beneficial for treating cancers with *ARID1A* mutations because of the compromised DNA damage repair [5]. Moreover, *ARID1A* mutations can act as a biomarker for assessing the effectiveness of new therapeutic strategies.

ARID1A is known to play a crucial role in controlling gene expression, primarily due to its ability to modulate the accessibility of chromatin to a variety of transcription factors through histone marks. The modulation of chromatin structure by ARID1A can have both positive and negative impacts on transcription [12]. Conventionally, histone acetylation, specifically at the H3K27ac site, results in an open chromatin configuration, promoting gene transcription. Conversely, histone methylation, notably at the H3K9me3 site, promotes a condensed chromatin state, thus inhibiting gene transcription. For instance, depletion of ARID1A leads to a reduction in the open histone mark (H3K27ac) at the gene enhancer regions, which in turn transcriptionally downregulates several cancer genes, such as *PIK3IP1* [13], *SLC7A11* [14], *CDKN1A*, *TGF-β* receptor [15], and *SMAD3* [16]. In contrast, ARID1A depletion can also upregulate the expression of certain genes (*USP9X, HDAC6, AURKA, TERT*) by promoting histone acetylation [17, 18]. Notably, ARID1A modulates RNA polymerase II (RNAPII) dynamics to regulate global transcription [19].

The mitogen-activated protein kinase (MAPK) signaling pathway is crucial for various cellular processes, such as proliferation, differentiation, migration, and survival. MAPK and ARID1A have been observed to have a negative correlation in endometrial carcinomas based on proteomics datasets from The Cancer Genome Atlas (TCGA). However, the exact mechanistic relationship between these two remains unclear. For example, in low-grade endometrial cancer, the MAPK pathway is predominantly activated, and concomitantly, ARID1A is often inactivated [20]*.* The MAPK pathway can also be activated by crosstalk with the activated PI3K/Akt pathway in ARID1A-deficient cancers or by upstream *KRAS* mutations [21–25]. In endometrial cancer, targeting the MAPK pathway has not yet reached an optimal point due to the development of therapeutic resistance [26, 27]. Targeting the MAPK pathway using its endogenous negative regulator could be an effective way to block the activated MAPK pathway [28]. One such negative regulator of the MAPK pathway is DUSP4, which induces the dephosphorylation of members belonging to the MAPK pathway [29–31]. Also referred to as mitogen-activated protein kinase phosphatase 2 (MKP-2), its low expression or downregulation is linked to aggressive tumor phenotypes, metastasis, and poor prognosis [32–34] [35], and an enhancer of chemotherapy efficacy [36–38]. Despite its importance, the role of DUSP4 has not been extensively studied in gynecologic cancer, especially in those with *ARID1A* mutations.

In this study, we utilized a unique endometrial epithelial cell line model to elucidate the relationship between ARID1A, DUSP4, and MAPK activation. We identified DUSP4 loss as an important mechanism of MAPK activation that may promote the tumorigenesis of human endometrial epithelial cells affected by *ARID1A* mutations. We also provide the first evidence that ARID1A protein controls the expression of DUSP4 by enhancing its promoter acetylation. A comprehensive understanding of the ARID1A-DUSP4-MAPK axis would be fundamental for designing an effective treatment or *ARID1A*-mutated cancers.

## Materials and methods

### Cell lines and culture conditions

The hEM3 cell line was created by introducing SV40-TAg via lentivirus to epithelial cells derived from healthy human endometrial tissue. Next, the *ARID1A* gene was deactivated in hEM3 using CRISPR/Cas9 [18, 39]. These hEM3 cells were kept in RPMI 1460 medium (Gibco, 11875093) supplemented with 15% FBS (Sigma, F4135), 1% Pen/Strep, and 1% NEAA. For experimental purposes, the hEM3 cells were moved to RPMI containing 10% FBS and 1% Pen/Strep (Gibco). MCF10a control and ARID1A-deficient cells (HD PAR-058 and HD 101–022, Horizon Discovery) were grown in DMEM/F12 medium enriched with 5% horse serum, 20 ng/ml EGF, 10 μg/ml insulin, 0.5 mg/ml hydrocortisone, 100 ng/ml cholera toxin, and 1% Pen/Strep. Both the standard HCT116 cells, their ARID1A-deficient versions (HD PAR-073 and HD 104–049, Horizon Discovery), and ES2 cells were cultivated in RPMI with 10% FBS and 1% Pen/Strep. Every cell line was inspected for mycoplasma prior to use and then re-evaluated bi-monthly using the Mycoplasma Detection Kit (ATCC, 30–1012 K).

### RNA sequencing and data analysis

Human endometrial epithelial cells (both ARID1A-expressing and ARID1A-deficient) were cultivated in both regular growth conditions and serum-starved settings (using standard growth media without FBS) for a duration of 24 h. Total RNA extraction was carried out with the Qiagen RNeasy Plus Mini Kit. The RNA integrity was evaluated via the Agilent 2100 Bioanalyzer RNA Nano Chip. RNA sequencing was conducted on the Illumina HiSeq2500 device by GeneWiz, Inc., utilizing a 2 × 100 bp paired-end high output V4 chemistry setup. Differential expression analysis was performed using Cuffdiff (ver. 2.2.1.3) to produce a list of differentially expressed genes (FDR < 0.05, Benjamini–Hochberg FDR correction) between the tested groups.

### Western blotting

Proteins were extracted using ice-cold RIPA buffer, which contained 50 mM Tris–HCl (pH 7.4), 150 mM NaCl, 1% NP-40, and 0.5% sodium deoxycholate. This lysis buffer was further enriched with Halt Protease inhibitor cocktail (Thermo Scientific) and PhosSTOP (Roche). After preparing the lysates, they were centrifuged at 12,000 rpm for a 12-min duration at 4 °C. Subsequently, the clear supernatants were run on 4–15% Mini-PROTEAN® TGX™ Precast Protein Gels (Bio-Rad) and then transferred onto 0.2 µm PVDF membranes. These membranes underwent a blocking process using 5% BSA for an hour at room temperature. Overnight incubation with primary antibodies (ARID1A, DUSP4, TNC, BMP4, and actin) was performed at 4 °C. On the following day, after washing the membranes three times with 0.1% TBST, they were exposed to a horseradish peroxidase (HRP)-linked secondary antibody for an hour at room temperature. Visualization of the blots was achieved with the Clarity™ Western ECL Blotting Substrate. All antibodies used were from CST (Cell Signaling Technologies, USA).

### Immunohistochemistry

Formalin-fixed and paraffin-embedded tissue sections were subjected to deparaffinization and rehydration. Antigen retrieval was accomplished by using DAKO Target Retrieval Solution, akin to citrate buffer at pH 6.0, or Trilogy, similar to EDTA at neutral pH. Endogenous peroxidase activity was quenched with 3% H_2_O_2_. These sections were incubated with antibodies overnight at 4 °C. The DAKO EnVision + System-HRP goat anti-rabbit or anti-mouse IgG and DAKO DAB + Substrate Chromogen System were utilized to visualize the immunostained sections. Hematoxylin was used for counterstaining nuclei. Cover slides were secured with Cytoseal 60. We utilized the following commercially available antibodies confirmed to work in immunohistochemistry: rabbit anti-Arid1a (Sigma), anti-DUSP4, anti-p-ERK, anti-p-P38, and anti-p-JNK (CST). The H-score system was implemented to assess immunoreactivity. This system involves determining the total scores from combined products of varied staining intensities on a semiquantitative scale ranging from 0 (no staining) to 3 (strong) and multiplying by the percentage of cells displaying positive reactions for each intensity. This approach yields a score for each sample that can vary between 0 and 300.

### cDNA synthesis and quantitative RT‒PCR

The first strand of cDNA was synthesized using an iScript cDNA synthesis kit (Bio-Rad). Then, a quantitative reverse transcription PCR (Polymerase Chain Reaction) process was performed using OneTaq® Hot Start DNA polymerase (New England Biolabs) and SYBR Green I (Life Technologies). The primers used for quantitative RT‒PCR are as follows:

**Table:** 

Genes	Primers (5′-3′)
ARID1A	F-CAG TAC CTG CCT CGC ACA TA
	R-GCC AGG AGA CCA GAC TTG AG
TGFBR2	F-CTG CAC ATC GTC CTG TGG
	R-GGA AAC TTG ACT GCA CCG TT
ACTB	F-GTT GTC GAC GAC GAG CG
	R-GCA CAG AGC CTC GCC TT
DUSP4	F-GCATCACGGCTCTGTTGAAT
	R-GCCTCACCCGTTTCTTCATC

### Chromatin immunoprecipitation assay

The chromatin immunoprecipitation (ChIP) assay was performed on two distinct clones of human endometrial epithelial *ARID1A*^WT^ and *ARID1A*^KO^ (*ARID1A*-/-) cells. These cells were cultured in 15 cm dishes, and ~ 1.2 × 10^7^ cells underwent cross-linking with Diagenode ChIP cross-link Gold and 1% formaldehyde as per the provided manufacturer's guidelines. The nuclear contents were isolated using the truChIP Chromatin Shearing kit (Covaris), adhering to the directions given by the manufacturer. For 12 min, chromatin was subjected to shearing in shearing buffer with a Covaris E220 focused ultrasonicator, ensuring that fragment sizes remained within the 200 to 600 bp range. The resulting sonicated lysates underwent a fivefold dilution using ChIP dilution buffer (0.1% Triton X-100, 2 mM EDTA, 20 mM Tris–HCl pH 7.5, 150 mM NaCl, and 1 × protease inhibitor) and then were immunoprecipitated with an overnight rotation at 4 °C with 0.5–3 µg of the following antibodies: Trimethyl-Histone H3 Lys27 (Millipore, #07-449), Trimethyl-Histone H3 Lys9 (Abcam, #ab8898), Acetyl-Histone H3 (Lys9) (CST, #9649), Acetyl-Histone H3 (Lys27) (CST, #8173). The antibody-chromatin complex was subsequently precipitated for 3 h using Protein A/G DYNAL magnetic beads (40 µl of 1:1 mixture). The bead-bound antibody-protein complexes underwent a series of washes: once with a low salt buffer, once with a high salt buffer, once using LiCl buffer, and twice with TE at pH 8.0. The DNA and protein complexes were digested in TE buffer containing 1% SDS, 200 mM NaCl, and 1 U of Proteinase K (Thermo Scientific) and heated at 56 °C for 2 h. Cross-linking reversal was achieved by heating the mix at 65 °C for 4 h. The DNA fragments were then purified with the QIAquick PCR Purification Kit, eluting in 55 µl of EB elution buffer. The JHMI Deep Sequencing and Microarray The core managed the Tru-seq ChIP-seq library preparation and sequencing using the NextSeq500 platform to produce single-end reads spanning 75 bases. A detailed description of ChIP-seq data processing can be found elsewhere [15]

### Ectopic expression of DUSP4

Human DUSP4 cDNA was cloned into the pCMV-Tag2B vector (Lifescience market, Model: PVT10712). The isogenic cell lines ES2-WT and ES2-AKO (*ARID1A* knockout) were transfected with vector only (pCMV-FLAG) or recombinant plasmid (pCMV-FLAG-DUSP4) using Lipofectamine 2000 (Invitrogen). The expression was detected by Western blotting.

### siRNA-mediated ARID1A knockdown and cell proliferation

*ARID1A* wild type ES2 cells were cultured and upon 70% confluence transfected with *ARID1A* siRNA pool (Dharmacon^™^, Cat. L-017263-00-0010) or non-targeting control siRNA pool (Dharmacon^™^, Cat. D-001810-10-05) using Lipofectamine™ RNAiMAX transfection reagent for 48 h. After transfection, cells were trypsinized and lysed for western blotting or counted and seeded (1000 cells/well in a 96-well format plate) to assess cell proliferation rate using CellTiter-Blue® reagent (Promega, Cat. G8080) after ARID1A knockdown.

### Animal studies: ES2 xenograft and ERKi treatment

ES2 or ES2 AKO cells (2 × 10^6^ per injection site) were mixed in Matrigel (Corning, 356234) and then subcutaneously injected into both sides of female Nu/Nu mice aged between 6 and 8 weeks. When the tumors reached an approximate volume of ~ 200 mm^3^, the mice were segregated randomly into two groups for each cell line (for ES2: ES2 UT and ES2 ERKi; for ES2 AKO: ES2 AKO UT and ES2 AKO ERKi). The treatment involved either a vehicle solution (5% DMSO in PBS) or 50 mg/kg ERKi (ulixertinib, Selleckchem, Catalog No. S7854), which was administered via intraperitoneal (i.p.) injections every other day over a span of three weeks. Tumor measurements started on the first day of the treatment and continued twice weekly. The same dosage of ulixertinib administered by the same route was used for the iPAD mouse model*.* Calipers were utilized for the consistent measurement of the tumor size. The volume of the tumor was calculated using the following equation: ${\text{TV}}\, = \,{4}/{3} \pi \left( {{\text{length}}/{2}} \right) \left( {{\text{width}}/{2}} \right)^{{2}}$. The study was concluded once the collective tumor size approached ~ 2000 mm^3^. Afterward, the mice were humanely euthanized, and the tumors were either preserved in formalin or stored at − 80 °C. Immunostained slide evaluations were conducted by a gynecological pathologist (IMS) who was unaware of the specific in vivo treatments. The use of animals was approved by the Institutional Animal Care and Use Committee (IACUC).

### Mouse models

*Arid1a*^*flox/flox*^ mice with a 129S1 genetic background and *Pten*^*flox/flox*^ mice with a BALB/c background (strain C;129S4-*Pten*^*tm1Hwu*^/J) were sourced from the Jackson Laboratory. The Cre-induced deletion in *Arid1a* removes exon 8, which results in a frameshift mutation leading to a premature stop codon (p.Gly809Hisfs*6) [40]. Similarly, the Cre-induced deletion in *Pten* removes exon 5 and introduces a frameshift mutation (p.Val85Glyfs*14) [41]. Mice with *Arid1a*^*flox/flox*^;*Pten*^*flox/flox*^ genotypes were produced by breeding these two transgenic lines. To specifically induce Cre recombinase expression in the mouse uterine epithelium for the described genetic modifications, we utilized *Pax8-Cre* mice. These were created by interbreeding mice that express the reverse tetracycline-controlled transactivator (rtTA) regulated by the Pax8 promoter (Pax8-rtTA) with another group of mice that express Cre recombinase in response to tetracycline (TetO-Cre) [42]. To generate the mouse models displaying either individual or combined knockout of *Arid1a* and *Pten* in the uterine epithelium, we bred *Pax8-Cre* mice with *Arid1a*^*flox*/flox^, *Pten*^*flox*/flox^, and *Arid1a*^*flox*/flox^;*Pten*^*flox*/flox^ strains. The initiation of the knockout process involved administering doxycycline to the mice, either via oral gavage at a dosage of 2 mg/mouse/day or by subcutaneously implanting 200 mg doxycycline pellets into the mice once they were 6–8 weeks old, marking puberty. Genotyping primers are listed elsewhere [15]. The Johns Hopkins University Animal Care Committee granted approval for all animal-related procedures.

### In silico proteomic analysis

Correlation data between the proteins ARID1A and MAPKs, as well as DUSP4 and MAPKs, was obtained from two databases: The Cancer Proteome Atlas (TCPA) (https://tcpaportal.org/tcpa/) and The Cancer Genome Atlas (TCGA) (https://tinyurl.com/4udmncxf). Reverse-phase protein array (RPPA) data from 423 samples from the Uterine Corpus Endometrial Carcinoma (TCGA, PanCancer Atlas) study were retrieved using cBioPortal. Additionally, RPPA data from 244 “MDACC endometrial carcinoma” samples and 404 samples from the “TCGA Uterine corpus endometrial carcinoma (UCEC)” were retrieved from TCPA, which is maintained by MDACC (MD Anderson Cancer Center). This comprehensive dataset enabled our analysis of the correlations among these proteins.

### Statistical analysis

Statistical significance between two groups was evaluated using unpaired Student’s *t* test (two-tailed) unless otherwise stated. Data are presented as the mean ± standard error of the mean (SEM). All individual experiments were performed in triplicate. Differences were considered significant if *p* < 0.05 (*), *p* < 0.01 (**), and *p* < 0.001 (***). All statistical analyses were conducted using the GraphPad Prism software.

## Results

### DUSP4 is downregulated in *ARID1A* knockout cells

**Fig. 1:**
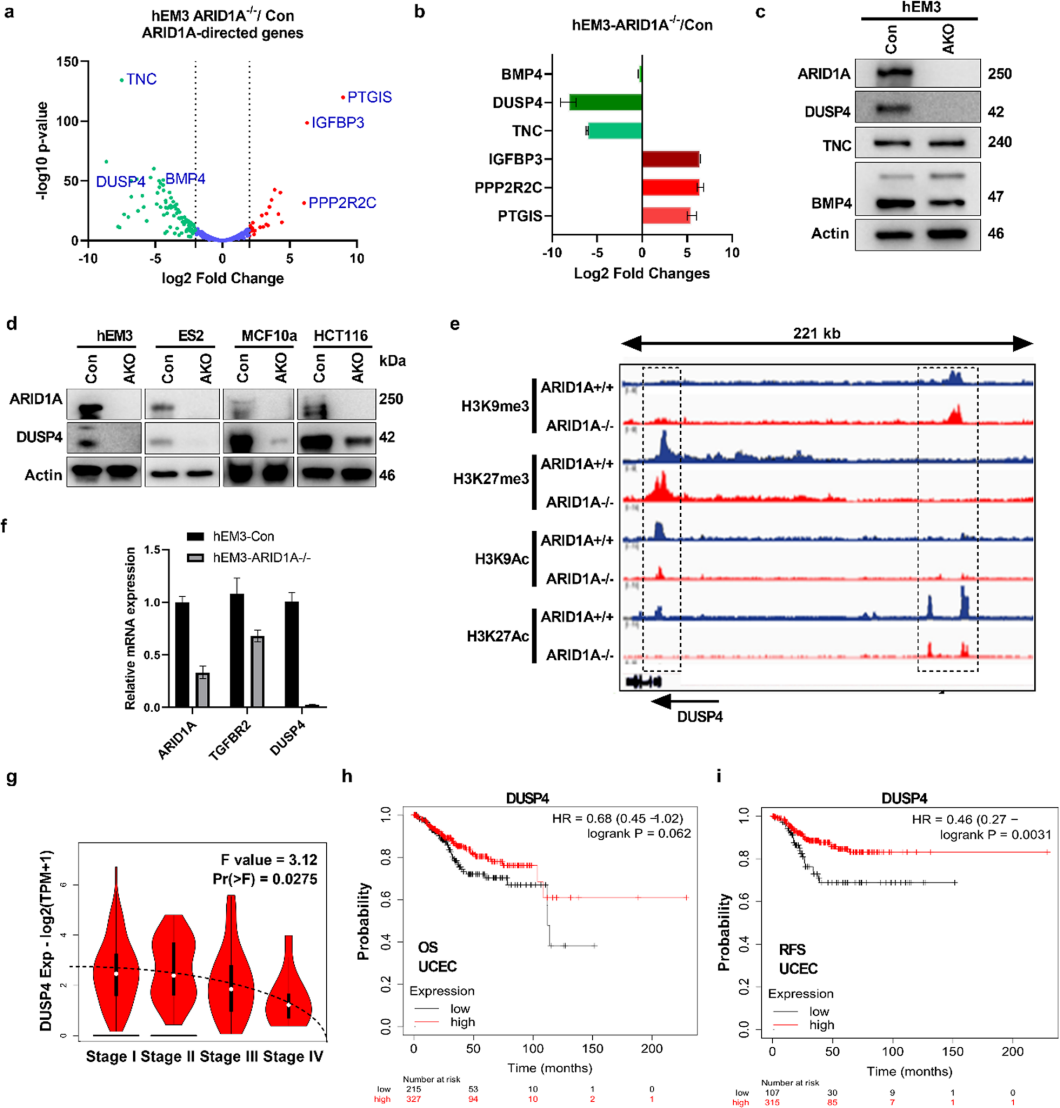
ARID1A regulates the expression of DUSP4. **a** Differential expression of genes using RNA sequencing between human endometrial epithelial cells (hEM3-Con) and *ARID1A* knockout cells (hEM3 *ARID1A*−/−). Prominent genes are shown in the graph in purple. **b** Fold changes of the indicated genes of interest discovered by RNA-seq are plotted. **c** Representative Western blot validating the indicated proteins in hEM3-Con and hEM3 ARID1A knockout (*ARID1A*−/−) cells. **d** Representative Western blot validation of DUSP4 downregulation after ARID1A loss in multiple isogenic cells. **e** The University of California Santa Cruz (UCSC) genome browser view of the occupancy of H3K9me3, H3K27me3, H3K9Ac, and H3K27Ac in the regulatory region of DUSP4. ChIP-seq tracks show peaks of methylation and acetylation histone marks on the DUSP4 promoter and enhancer regions. Dotted rectangles indicate locations of important binding events. The right dotted rectangle shows the DUSP4 enhancer region, whereas the left dotted rectangle indicates the promoter region. The arrow underneath the graph indicates the orientation of DUSP4 transcription. **f** qRT‒PCR validation of DUSP4 downregulation in ARID1A-deficient hEM3 cells (*ARID1A*−/−) compared to isogenic control cells. Measurement of ARID1A and TGFBR2 mRNA levels was performed as additional controls. TGFBR2 was previously reported as a direct target of ARID1A [15]. **g** DUSP4 mRNA expression across different stages of endometrial cancer (UCEC) with a sample size of N = 174. The dotted line indicates a decrease in DUSP4 expression as the disease progresses, (accessed via: http://gepia2.cancer-pku.cn/#analysis). **h** Kaplan‒Meier plot showing overall survival (OS) and **i** relapse-free survival (RFS) based on DUSP4 mRNA expression levels in UCEC (uterine corpus endometrial carcinoma) patients with a sample size of 543, (Accessed via: http://kmplot.com/analysis/index.php?p=service)

Somatic inactivating *ARID1A* mutations are prevalent in human endometrium-related malignancies. In our previous study, we reported that such deleterious mutations contribute to tumorigenesis by transcriptional reprogramming through its chromatin remodeling function [15]. However, specific signaling pathways or networks that are regulated by ARID1A in genetically deleted models remain to be determined. To this end, we revisited our RNA-sequencing (RNA-seq) data performed on isogenic cell pairs of human endometrial epithelial cells with wild-type *ARID1A* (designated as hEM3 con) or *ARID1A* gene deletion (designated as hEM3 *ARID1A*^−/−^). Among the prominent genes that are significantly downregulated in hEM3 *ARID1A*^−/−^ cells compared to hEM3 con cells are tenascin C (TNC), dual specificity phosphatase 4 (DUSP4), and bone morphogenetic protein 4 (BMP4). In contrast, prostacyclin synthase (PTGIS), insulin-like growth factor-binding protein 3 (IGFBP3), protein phosphatase 2 regulatory subunit B, and gamma isoform (PPP2R2C) are notable genes showing significant upregulation in hEM3 *ARID1A*^−/−^ cells compared to hEM3-Con cells (Fig. 1a, b).

These differentially expressed genes play context-dependent cancer-promoting or cancer-suppressing roles. We validated our genes of interest derived from the RNA-seq data using Western blotting (Fig. 1c) and qPCR (Fig. 1f). Although we did not observe significant downregulation of TNC, BMP4 protein levels decreased by approximately 50% after *ARID1A* was deleted by CRISPR-mediated KO (Fig. 1c). Interestingly, the mRNA and protein levels of DUSP4 were completely lost in hEM3 *ARID1A*^−/−^ cells. This observation was validated using additional isogenic cell line pairs (Fig. 1d). DUSP4 expression level decreases according advances in clinical stages (Fig. 1g). Low DUSP4 expression was indicative of poor overall survival (OS) and relapse-free survival (RFS) in endometrial cancer patients (Fig. 1h, i). In summary, DUSP4 was significantly downregulated at both the mRNA and protein levels after ARID1A loss, and as DUSP4 is a well-known negative regulator of the MAPK pathway, we focused on DUSP4 in our subsequent investigations and were interested in how ARID1A controls its expression.

### ARID1A alters histone acetylation in the DUSP4 regulatory region

To understand how ARID1A controls DUSP4 expression, we used ChIP sequencing to analyze the binding of ARID1A to the promoter and regulatory regions of DUSP4 and the densities of transcription repression histone marks (H3K9me3, H3K27me3) and active transcription histone marks (H3K9Ac, H3K27Ac) present in these regions. We used the isogenic cell lines hEM3-con and hEM3 ARID1A^−^^/^^−^ for the ChIP-seq assays. The ChIP-seq histogram (Fig. 1e) indicated that the repressive histone marks H3K9me3 and H3K27me3 are unchanged in the promoter and regulatory regions of the DUSP4 gene irrespective of ARID1A status. Conversely, the active histone marks (H3K9Ac, H3K27Ac) were significantly reduced in *ARID1A* knockout cells in both the distal (enhancer) and proximal region (promoter) regions of the DUSP4 gene. Moreover, RNA pol II binding was significantly reduced at these regions, indicating its regulation of DUSP expression together with histone acetylation [15]. Not only the reduction in acetylation marks but also the loss of RNA pol II and ARID1A in these regions may be responsible for the downregulation of DUSP4.

### ARID1A loss is positively correlated with DUSP4 loss in vivo and in human uterine endometrioid carcinoma tissues

**Fig. 2:**
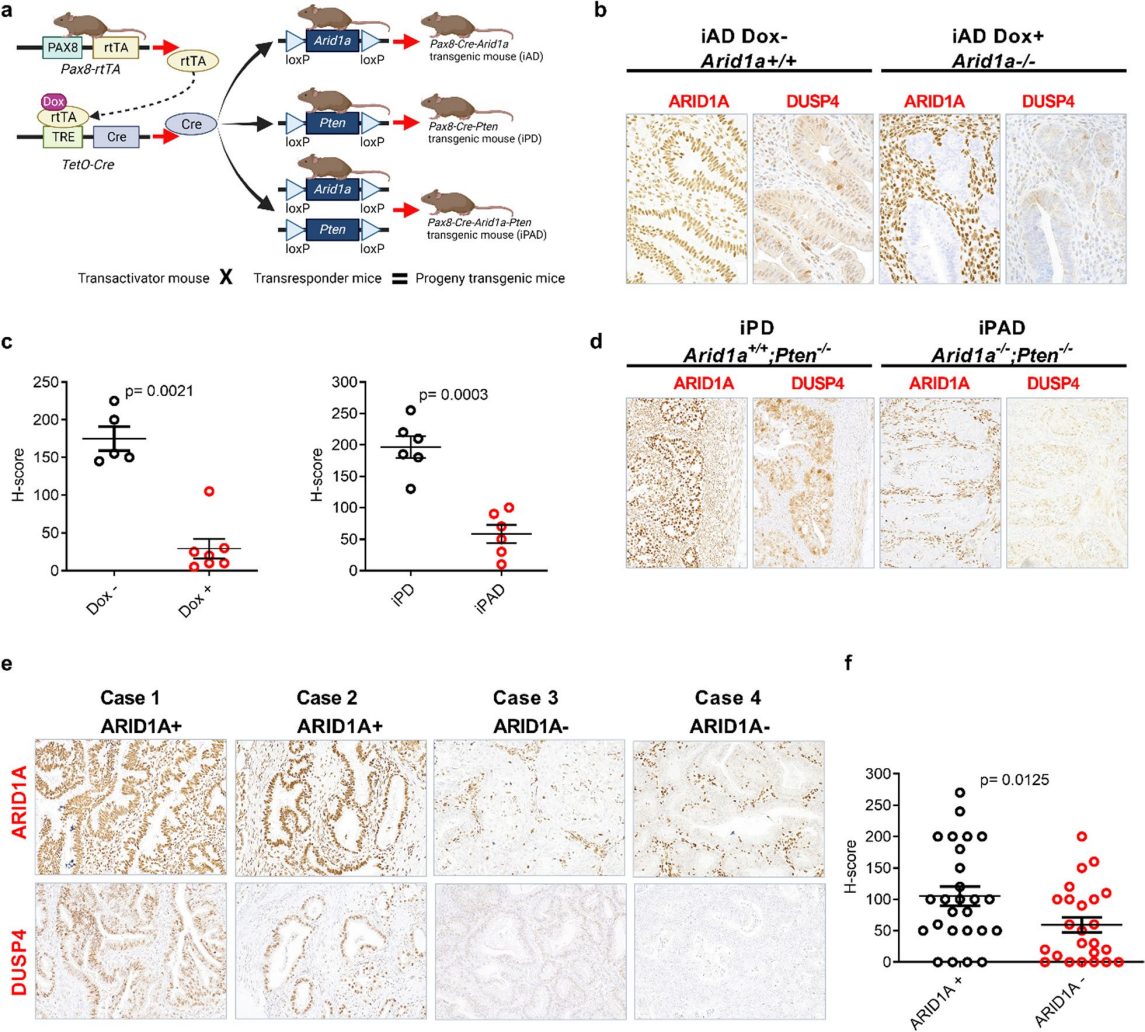
Correlation of ARID1A and DUSP4 expression in mouse endometrial tissues and human uterine endometrioid carcinoma tissues. **a** A schematic presentation of the genetically engineered mouse models used in this study. The deletion of *Arid1a* and/or *Pten* in Pax8-expressing tissues (uterine epithelial cells) is achieved by activation of the Pax8 promoter via administration of doxycycline (see sect. “Materials and methods”). **b** Representative photomicrographs of *Arid1a* and *Dusp4* immunoreactivity in iAD mice with (Dox +) or without (Dox−) doxycycline-induced *Arid1a* deletion. **d** Representative photomicrographs of *Arid1a* and *Dusp4* immunoreactivity in iPD and iPAD mouse models in the presence of doxycycline-induced gene deletion. **c** H-score quantitation of DUSP4 expression in iAD, iPD, and iPAD mice. iAD mice: n = 7 for the Dox + group, n = 5 for the Dox- group; iPD mice, n = 6; iPAD mice: n = 6. **e** Representative photomicrographs of ARID1A and DUSP4 immunoreactivity from human uterine endometrioid carcinoma tissues. **f** H-score quantitation of DUSP4 immunoreactivity in human uterine endometrioid carcinoma tissues. *iAD* inducible Arid1a deletion, *iPD* inducible Pten deletion, *iPAD* inducible *Arid1a* and *Pten* deletion, *Dox−* mice did not receive doxycycline, *Dox + * mice received doxycycline, *ARID1A*^*+*^ ARID1A retained, *ARID1A*^*−*^ ARID1A loss. A P < 0.05 was considered significant in (**c**) and (**f**)

Next, we utilized a genetically engineered mouse model named iPAD (*Arid1a, Pten, Pax8-Cre*) with conditional deletion of *Arid1a*, *Pten,* or both in *PAX8*-expressing uterine epithelium by doxycycline application. Two control mouse models, iAD and iPD, with conditional *Arid1a* or *Pten* single gene deletion were also generated (Fig. 2a). The expression of DUSP4 was first evaluated in iAD mice with or without *Arid1a* deletion*.* The immunoreactivity of mouse ARID1A and DUSP4 proteins was positively correlated; when *Arid1a* was deleted in epithelial cells, DUSP4 was also lost (Fig. 2b, 2c). The immunoreactivity of DUSP4 is plotted as an H-score (Fig. 2c). In contrast, the expression of DUSP4 remained high in the iPAD mouse uterine epithelium even when *Pten* was deleted (Fig. 2d). Based on the H-score to semi-quantify staining pattern, we showed that in the iAD model, DUSP4 levels were significantly lower in *Arid1a* single gene knockout (+ Dox) mice than in *Arid1a* gene-retained mice (−Dox) (Fig. 2c, p = 0.0003) and intact *Arid1a* (iPD) mice (Fig. 2d, c). Although it can be argued that *Pten* deletion in the iPAD model could contribute to this result, we also showed the same result using the *Arid1a*-only deletion model (iAD) (Fig. 2b).

To further strengthen this finding, we obtained uterine endometrioid carcinoma tissues and segregated them into ARID1A-high and ARID1A-low groups by immunoreactivity. ARID1A-low group had significantly lower DUSP4 expression (p = 0.0125) in carcinoma tissues (Fig. 2e, f). In summary, using genetically engineered mouse models, we demonstrated that ARID1A but not PTEN controls the expression of DUSP4.

### ARID1A loss activates MAPK proteins that are the targets of DUSP4 dephosphorylation

**Fig. 3:**
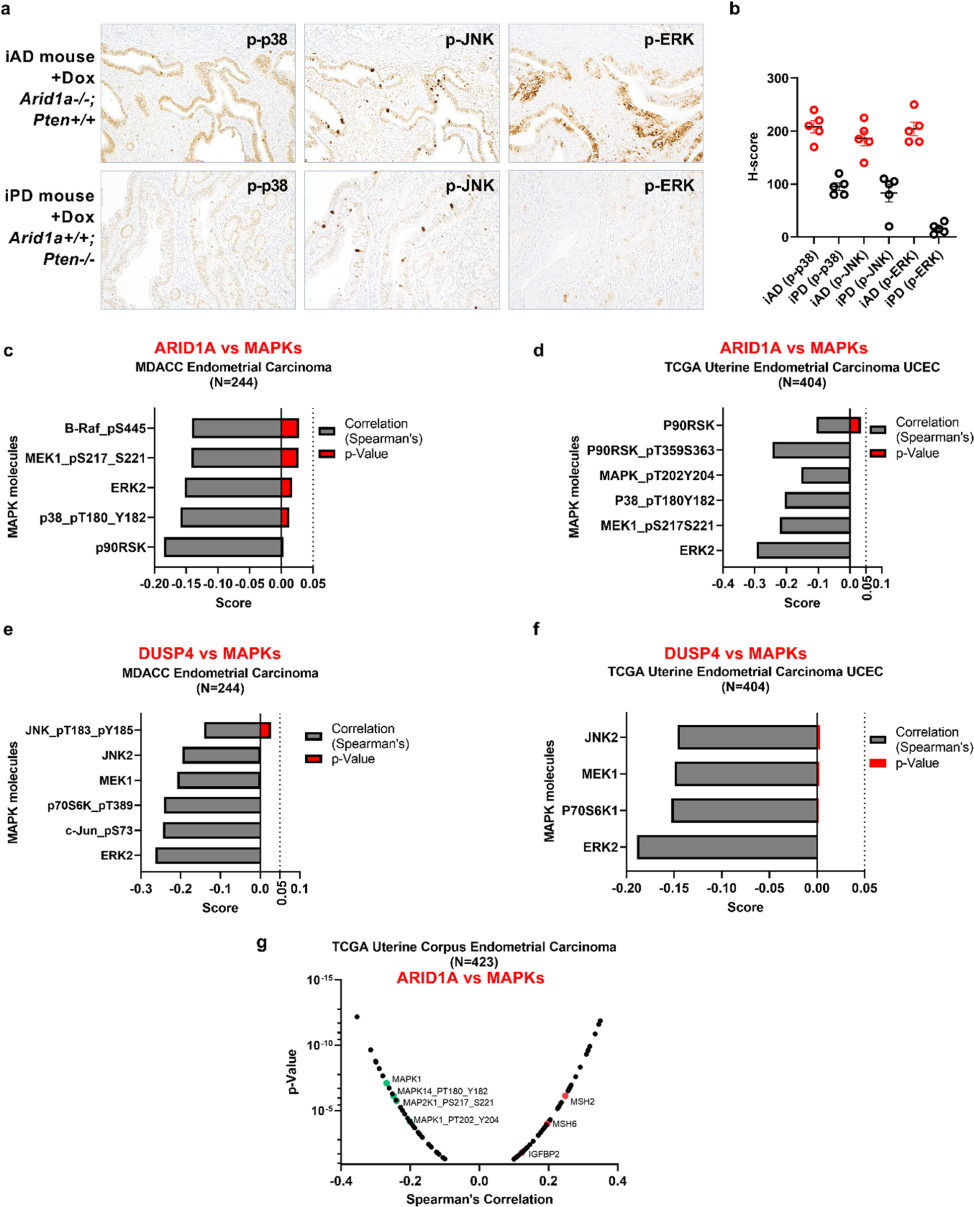
Correlation of ARID1A, DUSP4, and MAPKs expression levels in vivo and in silico: **a** Representative photomicrographs of MAPKs (p-p38, p-JNK, and p-ERK) immunoreactivity in iAD and iPD mice in the presence or absence of doxycycline-induced gene deletion*.* **b** H-score semi-quantification of MAPK immunoreactivity in iAD and iPD mice. **c**, **d**, **g** Spearman’s correlation between ARID1A and MAPKs in the three indicated datasets. **e**, **f** Spearman’s correlation between DUSP4 and MAPKs in the two indicated datasets. In **c**–**g**, the red bar indicates the p-value (p < 0.05 is considered significant). **c**–**f** Data were retrieved from the TCPA website (https://tcpaportal.org/tcpa/) **g** Data were retrieved from TCGA through cBioPortal (https://www.cbioportal.org/)

Next, we investigated whether ARID1A loss-mediated downregulation of DUSP4 upregulated the targets of DUSP4, including MAPKs: p-ERK, p-p38, and p-JNK [30, 43–45]. To this end, we utilized our model and mined proteomics databases for correlation studies. As shown in Fig. 3a, *Arid1a* loss (iAD mouse) was associated with the upregulation of all targets of DUSP4 (p-ERK, p-p38, and p-JNK), whereas immunoreactivity was significantly reduced when *Arid1a* was intact (iPD mouse) (Fig. 3a, 3b). Similarly, we explored human proteomics databases incuding the Clinical Proteomic Tumor Analysis Consortium (CPTAC) to correlate the expression between ARID1A and DUSP4, as well as MAPK molecules. As expected, expression of MAPK signaling molecules (B-Raf_pS445, MEK1_pS217_S221, ERK2, p38_pT180, and p90RSK) were negatively correlated with ARID1A expression (Fig. 3c) in the “MDACC endometrial carcinoma” dataset. In the same dataset,  we also found that expression of MAPK signaling molecules (JNK_pT183_pY185, JNK2, MEK1, p70S6K_pT389, c-Jun_pS73, and ERK2) were negatively correlated with DUSP4 expression (Fig. 3e). A similar observation was also seen in the TCGA dataset (Fig. 3d, f). The same conclusion was also made from the TCGA database for proteomics (Fig. 3g, Additional file 1: Fig. S1). In summary, expression of  MAPKs was negatively correlated with the expression of ARID1A and DUSP4.

### Overexpression of DUSP4 suppresses ES2 cell proliferation in vitro

**Fig. 4:**
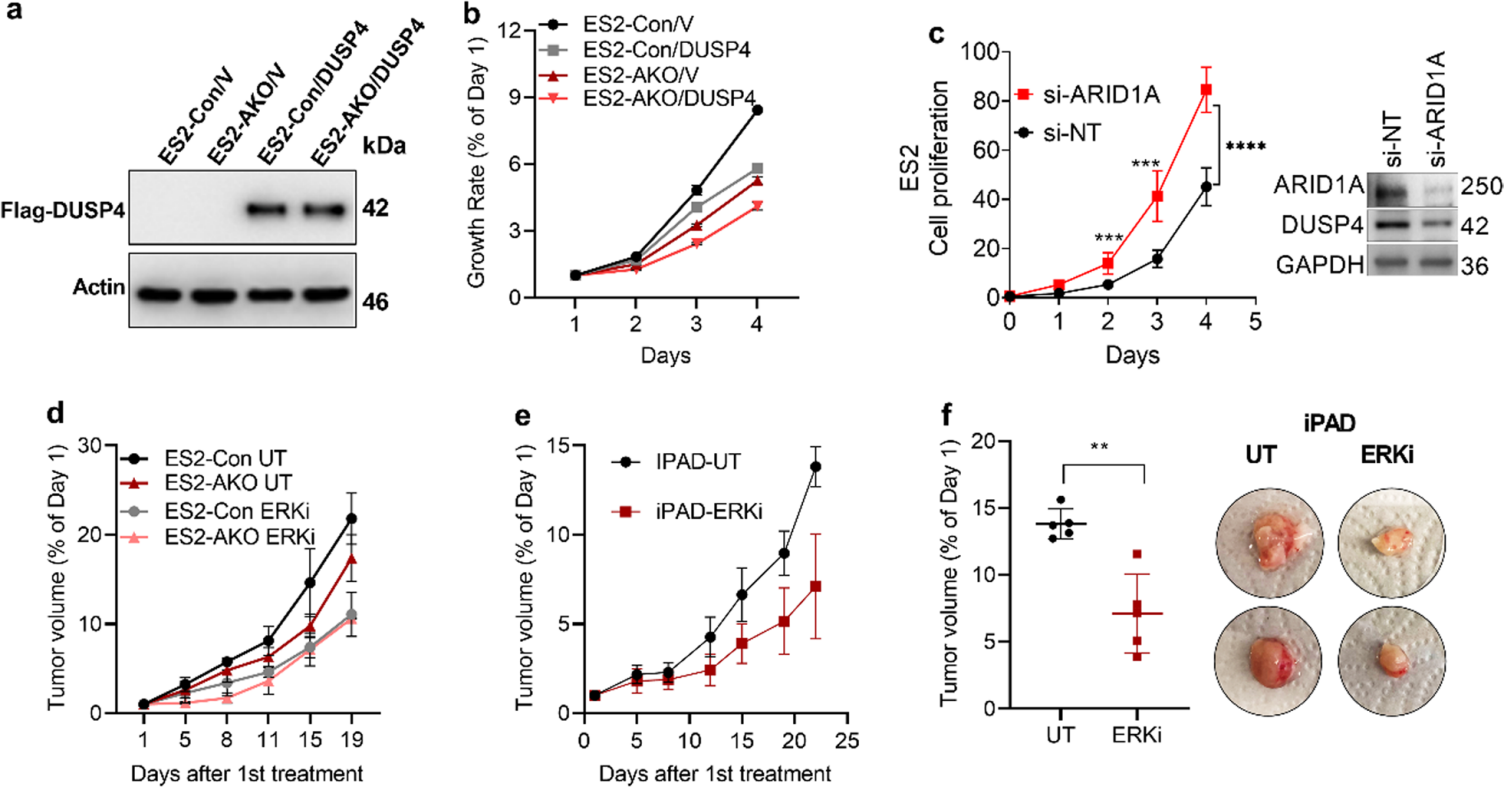
The effects of ectopic DUSP4 expression in vitro and in vivo.  **a** Confirmation of DUSP4 overexpression by Western blotting in ES2 cells with (ES2-Con) or without ARID1A (ES2-AKO). **b** Measurement of the cell proliferation rate using CellTiter-blue after DUSP4 transfection. **c** Measurement of the cell proliferation rate in ES2 cells after siRNA mediated ARID1A knockdown. Western blot panel shows the knockdown efficiency of ARID1A siRNA pool and positive correlation ARID1A with DUSP4 protein expression. siNT denotes control non-targeting siRNA whereas si-ARID1A targeted ARID1A. GAPDH expression was used as a loading control. **d** Tumor volume measurement graph of ES2 isogenic cell xenografts in nude/nude mice with (ERKi) or without (UT) ERK inhibitor treatment for 19 days. **e** Antitumor effect of ERKi (ERK inhibitor) on iPAD mice. **f** Representative photomicrographs of tumor volume/size and quantitative analysis of tumor volume at the endpoint between the control (UT) and ERKi inhibitor-treated groups. Mice in the UT control cohort were administered PBS/DMSO. Data are presented as the mean ± SD

It has been established that DUSP4 negatively regulates the MAPK signaling to modulate various cellular processes, including cell proliferation. To study the effect of ectopic DUSP4 expression, we utilized another cell line, ES2, which was derived from human ovarian clear cell carcinoma, a tumor type related to endometriosis. *ARID1A* mutation is also prevalent in this cancer type. We transfected ES2 isogenic cells, ES2-Con (ARID1A-WT control) and ES2 AKO (ARID1A-knockout) cells either with an empty vector (ES2-Con/V; ES2-AKO/V) or a DUSP4 plasmid (ES2-Con/DUSP4; ES2-AKO/DUSP4). Validation of transfection was shown by Western blotting (Fig. 4a). When DUSP4 was overexpressed in ES2 cells with intact ARID1A, the cell growth rate was slowed, likely because of low MAPK activity in these cells (Fig. 4b). A similar observation was also seen in the case of ARID1A knockout cells (Fig. 4b). Despite the anticipation of higher cell growth rates in ES2-AKO cells compared to ES2 WT cells, it is important to consider that these cells may have varying ages and passage numbers. However, the positive aspect is that overexpression of DUSP4 can decrease the cell growth rate regardless of the ARID1A status, and this observation is in line with DUSP4 being a negative regulator of the MAPK pathway . To maintain a consistent passage number for a fair comparison of cell proliferation between ARID1A-proficient vs ARID1A-deficient ES2 cells, we opted for siRNA-mediated ARID1A knockdown approach. Following this approach, a significant increase in the cell proliferation rate was observed in the ARID1A-deficient cells starting from day 2, as depicted in Fig. 4c.

### In vivo targeting of ERK in ARID1A-deleted tumors slows tumor progression.

Our data presented above show that ARID1A loss downregulates DUSP4, a negative regulator of MAPKs. Therefore, pharmacological targeting MAPKs in vivo should slow tumor progression. Therefore, we used two models: a genetically engineered iPAD model (15), which recapitulates human endometrial cancer, and an ES2 xenograft model to evaluate the ERK targeting strategy. In the ES2 xenograft model, ERK inhibition using ulixertinib decelerated tumor development (Fig. 4c). ES2-Con xenograft mice that received ERKi treatment (cohort: ES2 Con ERKi) exhibited a decelerated rate of tumor development compared to that of the untreated cohort, ES2 Con UT. Additionally, when *ARID1A* KO ES2 tumors were treated with ERKi (cohort: ES2 AKO ERKi), a similar reduction in tumor growth was observed compared to the untreated cohort (ES2 AKO UT) (Fig. 4c). Targeting ERK with ulixertinib suppressed tumor development (Fig. 4d) and resulted in significantly smaller tumors (Fig. 4e, f) in the iPAD mouse model. In conclusion, these results suggest that targeting ERK holds promise as a therapeutic strategy for treating *ARID1A*-mutated cancers.

## Discussion

**Fig. 5:**
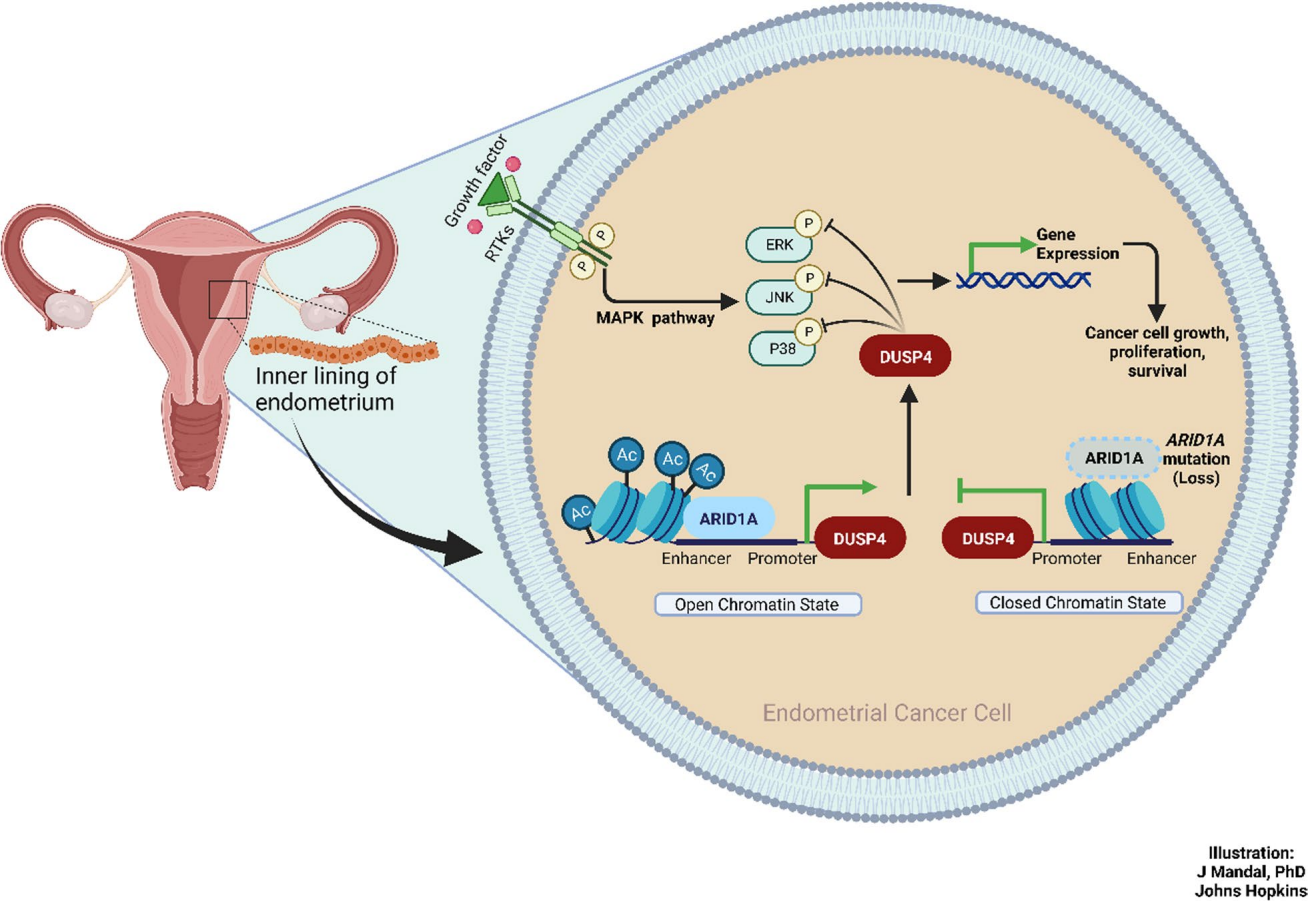
Simplified model illustrating ARID1A’s function in rmodulating MAPK activation through DUSP4 in endometrium-related cancers. The ARID1A protein binds to the regulatory DNA motifs (such as promoters and enhancers) of various genes, including *DUSP4*. ARID1A sustains active transcriptional histone marks, specifically acetylation. This facilitates an open chromatin state, allowing for the transcription of DUSP4. Notably, DUSP4 serves as a negative regulator of the kinases in the MAPK pathway. When ARID1A is lost, the chromatin transits to a closed state, leading to a decrease in DUSP4 levels and the subsequent activation of the MAPK signaling pathway

The overarching objective of our study was to delineate the mechanistic underpinning of *ARID1A* mutation-driven tumorigenesis . The main findings of this study include new evidence showing transcriptional regulation of DUSP4 by ARID1A (see Illustration in Fig. 5). We found that *DUSP4* was significantly downregulated in ARID1A-knockout hEM3 cells compared to parental hEM3 cells. ChIP-seq analysis further revealed that ARID1A bound and regulated active transcription histone marks in the regulatory regions of *DUSP4*, suggesting direct transcriptional control. This finding was further corroborated in our in vivo studies, wherein ARID1A loss was correlated with DUSP4 loss in the uterine epithelium of genetically engineered model mice and in human uterine endometrioid carcinoma tissues. Notably, we identified that ARID1A loss results in the upregulation of MAPKs, which are the targets of DUSP4 dephosphorylation. The result was confirmed through proteomics database exploration, revealing negative correlations between ARID1A, DUSP4, and MAPK molecules in large published datasets of endometrial carcinomas. The potential therapeutic implication of this finding is highlighted in our in vivo antitumor efficacy studies, in which ERK targeting by a small molecule inhibitor significantly reduced tumor growth in both iPAD, a genetically engineered murine model, and ES2 xenograft mouse model. To interpret our findings in simple terms, when ARID1A expression is lost, DUSP4 expression diminishes, consequently activating the MAPK pathway. This finding may represent another key mechanism through which *ARID1A* mutations promote cancer development.

The involvement of ARID1A in tumorigenesis through transcriptional reprogramming is well established in the literature [15], yet our research uniquely emphasizes its role in modulating MAPK pathway activation. Our findings align with previous studieshighlighting the importance of tumor suppressor function of DUSP4 in human malignancies [30, 32, 33, 35]. Although DUSP4 expression can be detected in patients with various malignancies, including endometrial cancer, its levels decrease in more advanced stages of the disease, suggesting that the MAPK pathway is activated in patients in advanced cancer stages.

There are several translational implications in this study. First, loss of ARID1A through genetic or epigenetic inactivation, as occurs frequently in human cancers, contributes to tumorigenesis [21, 46]. The use of multiple model systems, including isogenic cell lines, genetically engineered mouse models, xenograft models, and human tissue samples, offers robustness to the conclusions. To extrapolate our finding to human samples, we validated it in independent large proteomics databases showing correlations between ARID1A, DUSP4, and MAPKs. DUSP4, a well-established negative regulator of the MAPK pathway, might serve as a potential prognostic biomarker for endometrial malignancies, given its inverse correlation with disease progression and its association with overall survival. Moreover, the current study uncovers a potential ERK targeting strategy in cancers with ARID1A loss.

While compelling evidence is provided in this report regarding the regulatory interplay between ARID1A, DUSP4, and the MAPK pathway, further mechanistic studies could enhance the understanding of the specific interactions between ARID1A and DUSP4 and their subsequent effect on the MAPK pathway. For example, the exact molecular machinery by which ARID1A influences the epigenetic landscape, particularly the dynamics of histone acetylation marks at the DUSP4 regulatory regions, needs a more in-depth investigation. We primarily focused on DUSP4 as our gene of interest, but other genes that are differentially expressed in ARID1A^KO^ cells might also play a role in regulating the MAPK pathway. Moreover, although the use of mouse models and human tissue samples substantiates our findings, a more comprehensive analysis of clinical data could clarify the clinical significance. It would be of particular interest, for instance, to examine whether patient outcomes vary depending on their ARID1A and DUSP4 expression levels. This can be an aspect to investigate in future studies.

## Conclusions

Our work uncovers a novel mechanism by which ARID1A regulates DUSP4 expression and subsequent MAPK activity, highlighting the diverse mechanisms how ARID1A protein controls tumor development.  However, additional research is needed to further elucidate the signaling pathway cross talks orchestrated by *ARID1A*  in endometrial cancer and to determine the clinical benefit by targeting the ARID1A-DUSP4-MAPK axis. 

### Supplementary Information

**Additional file 1: Fig. S1.** Correlation between ARID1A and MAPK signaling activation in a TGCA endometrial cancer proteome study (https://www.cbioportal.org/). **a**–**d** Indicated MAPK molecules show significant negative correlation with ARID1A. This is the expanded form of the molecules from Fig. 3g

## Data Availability

Not applicable.

## Abbreviations

ARID1A: AT-rich interaction domain 1A
MAPK: Mitogen-activated protein kinase
DUSP4: Dual specificity phosphatase 4
PTEN: Phosphatase and tensin homolog
rtTA: Reverse tetracycline-controlled transactivator
SWI/SNF: Switch/sucrose non-fermentable
Pax8: Paired box 8
RPPA: Reverse-phase protein array
TCGA: The Cancer Genome Atlas
TCPA: The Cancer Proteome Atlas
UECE: Uterine corpus endometrial carcinoma;
PIK3IP1: Phosphoinositide-3-kinase interacting protein 1
SLC7A11: Solute carrier family 7 member 11
CDKN1A: Cyclin dependent kinase inhibitor 1A
TGF-β receptor: Transforming growth factor-beta receptor
SMAD3: SMAD family member 3
USP9X: Ubiquitin specific peptidase 9 X-linked
HDAC6: Histone deacetylase 6
AURKA: Aurora kinase A
TERT: Telomerase reverse transcriptase
ERK: Extracellular signal-regulated kinase
JNK: C-jun N-terminal kinase
p38: p38 mitogen-activated protein kinase
