# Supplementary material for: ARID1A loss activates MAPK signaling via DUSP4 downregulation
Source: J Biomed Sci. 2023 Dec 9;30:94. doi: 10.1186/s12929-023-00985-5 (PMC10709884; doi:10.1186/s12929-023-00985-5)
Supplement: Supplementary file 1 — Additional file 1: Fig. S1. Correlation between ARID1A and MAPK signaling activation in a TGCA endometrial cancer proteome study (https://www.cbioportal.org/). a–d Indicated MAPK molecules show significant negative correlation with ARID1A. This is the expanded form of the molecules from Fig. 3g [file 12929_2023_985_MOESM1_ESM.docx]

**ARID1A Loss Activates MAPK Signaling via DUSP4 Downregulation: A Mechanistic Insight in Endometrial Carcinoma**

**Additional file Figure S1**

~~
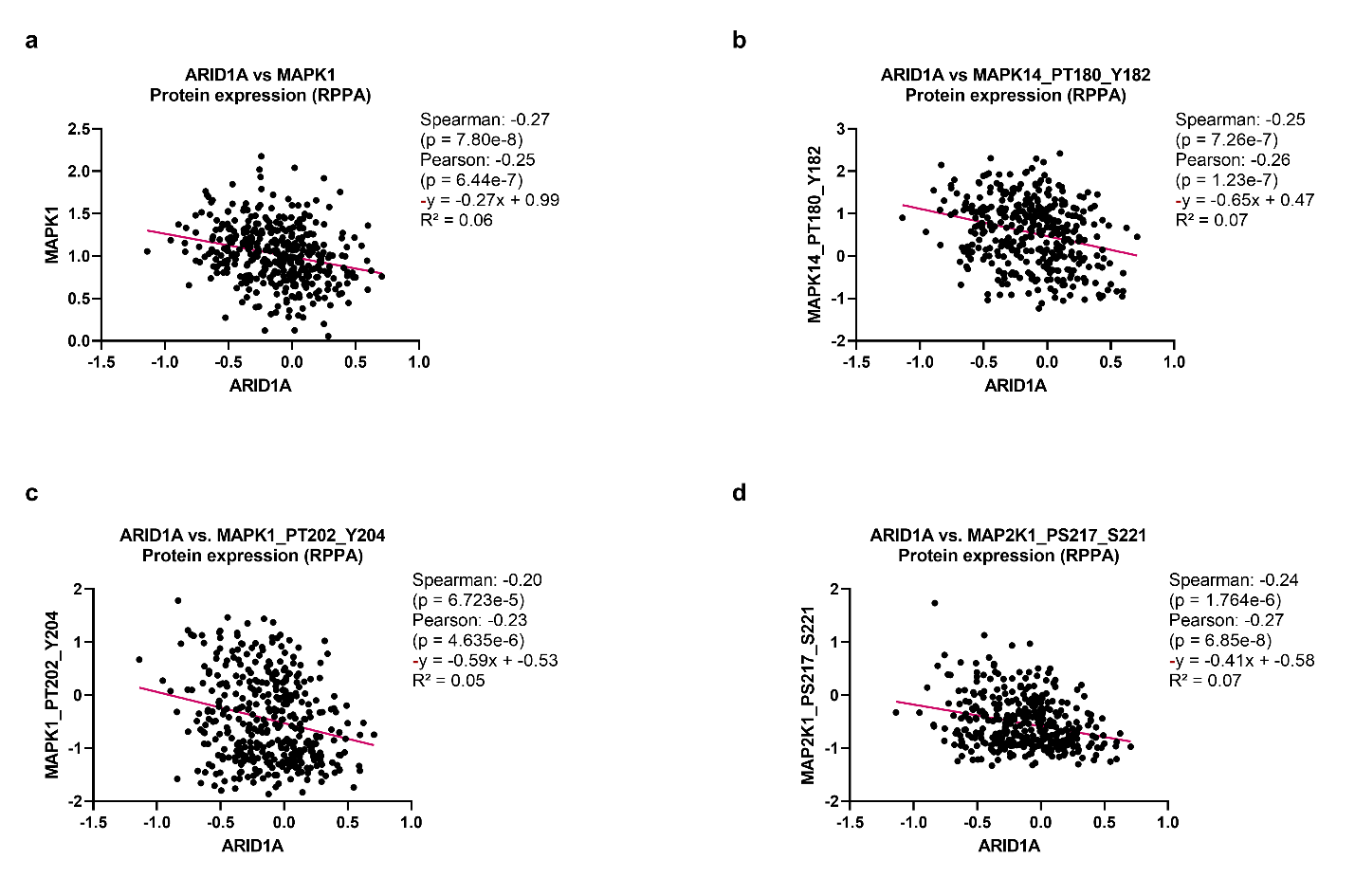
~~

**Fig. S1.** **Correlation between ARID1A and MAPK signaling activation in a TGCA endometrial cancer proteome study (**[**https://www.cbioportal.org/**](https://www.cbioportal.org/)**).** (a-d) Indicated MAPK molecules show significant negative correlation with ARID1A. Note: This is the expanded form of the molecules from fig3. g
